# Implementing a hypertension management programme in a rural area: local approaches and experiences from Ba-Vi district, Vietnam

**DOI:** 10.1186/1471-2458-11-325

**Published:** 2011-05-17

**Authors:** Quang Ngoc Nguyen, Son Thai Pham, Viet Lan Nguyen, Stig Wall, Lars Weinehall, Ruth Bonita, Peter Byass

**Affiliations:** 1 Department of Cardiology, Hanoi Medical University, 1 Ton-That-Tung Street, Dong-Da District, Hanoi, Vietnam; 2 Vietnam National Heart Institute, Bach Mai Hospital, 78 Giai-Phong Avenue, Dong-Da District, Hanoi, Vietnam; 3 Epidemiology and Global Health, Department of Public Health and Clinical Medicine, Umeå University, SE-90187 Umeå, Sweden; 4 School of Population Health, Faculty of Medical and Health Sciences, University of Auckland, Auckland 1142, New Zealand

## Abstract

**Background:**

Costly efforts have been invested to control and prevent cardiovascular diseases (CVD) and their risk factors but the ideal solutions for low resource settings remain unclear. This paper aims at summarising our approaches to implementing a programme on hypertension management in a rural commune of Vietnam.

**Methods:**

In a rural commune, a programme has been implemented since 2006 to manage hypertensive people at the commune health station and to deliver health education on CVD risk factors to the entire community. An initial cross-sectional survey was used to screen for hypertensives who might enter the management programme. During 17 months of implementation, other people with hypertension were also followed up and treated. Data were collected from all individual medical records, including demographic factors, behavioural CVD risk factors, blood pressure levels, and number of check-ups. These data were analysed to identify factors relating to adherence to the management programme.

**Results:**

Both top-down and bottom-up approaches were applied to implement a hypertension management programme. The programme was able to run independently at the commune health station after 17 months. During the implementation phase, 497 people were followed up with an overall regular follow-up of 65.6% and a dropout of 14.3%. Severity of hypertension and effectiveness of treatment were the main factors influencing the decision of people to adhere to the management programme, while being female, having several behavioural CVD risk factors or a history of chronic disease were the predictors for deviating from the programme.

**Conclusion:**

Our model showed the feasibility, applicability and future potential of a community-based model of comprehensive hypertension care in a low resource context using both top-down and bottom-up approaches to engage all involved partners. This success also highlighted the important roles of both local authorities and a cardiac care network, led by an outstanding cardiac referral centre.

## Background

Cardiovascular diseases (CVD) and their risk factors are emerging as the leading causes of mortality and morbidity in developing countries including Vietnam [1]. In 2002 it was estimated that one in three of all deaths were attributed to CVD [2]. Typically using educational or environmental changes to promote and facilitate changes in health behaviour, community-targeted interventions have attempted to modify the prevalence of one or more CVD risk factors (including hypertension) within specifically identified circumscribed communities, to reduce the overall CVD burden and improve population health outcomes [3,4]. "Four distinct generations" of community-level CVD intervention programmes over the last five decades demonstrated the value and effectiveness of population-based approaches, particularly when addressing specifically targeted communities or sub-populations [5-7]. However, the evidence reveals significant gaps in knowledge for delivering, implementing and maintaining effective community-targeted CVD prevention programmes [6,8-10].

Suffering from the 'double burden' of previously common communicable diseases and emerging non-communicable diseases during the early stages of health transition, rural people in Vietnam are witnessing the increasing prevalence and burden of hypertension as well as other CVD risk factors even in disadvantaged subpopulations, such as women of lower occupational or economic status [11-14]. Focusing on hypertension and behavioural CVD risk factors, a programme on hypertension management has been implemented since 2006 in Phu-Cuong commune, in a typical rural area of Vietnam, 60 km to the west of Hanoi city. Using the preliminary results from Phu-Cuong model after 17 months of implementation, this paper aims at summarising our approaches on how to implement such a programme and to involve all related partners, and finding potential factors which could influence local people's adherence to the management programme.

## Methods

**Figure 1:**
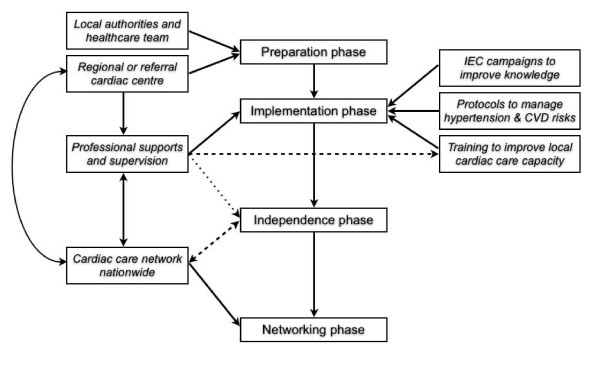
**Process to set up and develop a programme on hypertension management in adults at commune-level**.

The programme on hypertension management had been set up since 2006 at Phu-Cuong commune (Ba-Vi district, Ha-Tay province, Vietnam). The commune management programme consisted of four sequential phases (preparation, implementation, independence and networking phases), gradually reducing the supervision and support from the responsible regional cardiac centre (Vietnam National Heart Institute - VNHI) (Figure 1). In December 2006 (preparation phase), a cross-sectional survey covering a random sample of 1,200 adults (≥ 25 years old) was carried out to investigate the prevalence of hypertension and other CVD risk factors and to recruit people with high blood pressure (BP) to the management programme. During the subsequent implementation phase, local inhabitants with opportunistically detected high BP were also recruited to the programme. Our programme on hypertension management at commune level included three mutual interactive core components: (1) comprehensive information education and communication (IEC) campaigns to improve knowledge of CVD risk factors (including hypertension) for the entire commune; (2) standard protocols at the commune health station to routinely diagnose and treat hypertensive patients with multi drug therapy and lifestyle modification; (3) continuous training programme to improve the capacity of the local cardiac care team (including on-site hands-on training) (Figure 1). Using personal medical records at regular check-ups on fixed dates every month, the management programme started in July 2007 (implementation phase) to monitor people's BP, their CVD risk factors, their treatment adherence and any incident medical events. During monthly check-ups, existing or suspected hypertensives were re-invited in order to: re-measure their BP, re-adjust their prescription and lifestyle modification, receive their medications, re-assess any major adverse cardiac events (MACE) or adverse drug reactions (ADR), and to re-enforce their knowledge of hypertension and other CVD risk factors. In any visit, blood pressure was measured twice, one minute apart, with participants in a sitting position after 5 minutes of rest, following a standardized protocol using automatic sphygmomanometers (OMRON Healthcare Inc.^®^, Bannockburn, Illinois) with appropriately sized cuffs. A third measurement was performed if the difference between the first two measurements was more than 10 mmHg. People with systolic blood pressure (SBP) ≥ 140 mmHg and/or diastolic blood pressure (DBP) ≥ 90 mmHg and/or receiving any kind of antihypertensive treatment were diagnosed as cases of hypertension or high blood pressure. Hypertension was classified into 3 stages following the definition of WHO-ISH/JNC-VI [15,16]. Previously hypertensive people having BP below 140/90 mmHg, achieved by any kind of antihypertensive treatment, were considered as having well-controlled blood pressure. The programme at Phu-Cuong completed its preparation and implementation phases in December 2008, by which time local cardiac care teams independently and effectively managed local patients at the commune health station with minimum supervision from VNHI.

Data were collected from all individual medical records during 17 months of the implementation phase, including age, sex, number of self-reported behavioural CVD risk factors (including unhealthy diet: excessive salt, high fat or low fruit and vegetable consumption; heavy drinking; current smoking; low physical activity; overweight or obesity), personal and family history of CVD or chronic diseases, systolic and diastolic BP, number of daily tablets of antihypertensive drugs, any MACE (such as death, stroke, heart failure, acute myocardial infarction, hospitalisation due to cardiac problems), ADR or minor drug side effects. Available demographic data (including education status, marriage status, occupation, health insurance, distance to commune health station) in the screening survey were linked to follow-up data.

People with high BP after screening were divided into two groups: one group comprising people who joined the programme and one group with people who did not join the programme. These two groups, and a third group of new people who joined the programme later (opportunistic screening) were compared to detect potential factors which influenced local people's decisions to participate in the management programme.

Adherence to the hypertension management programme was measured by the number of check-ups (clinic visits) for each participant during the 17 month follow-up period, classified into three groups: regular follow-up (one check-up per one to two months on average), irregular follow-up (one check-up per three to six months on average), and drop-out (less than one check-up per six months). These three groups were compared to detect potential factors that influenced local people's decisions to leave the management programme.

Both descriptive and analytical statistical analyses were carried out using Stata10 software (StataCorp LP, Texas, USA). Means with standard deviations and proportions of variables of interest were calculated. Multivariate logistic regression modelling was performed to examine the probability of joining or leaving the programme in relation to several variables of interest. A significance level of p < 0.05 was used.

This study protocol was approved by the Scientific Ethical Committee in Biomedical Research of Hanoi Medical University (HMU) and the Ethical Committee of the Health System Research Project (HSRP) of HMU. All human subjects in the study were asked for their consent before collection of data, and all had complete rights to withdraw from the study at any time without any threat or disadvantage.

## Results

Both top-down and bottom-up approaches were applied to implement the hypertension management program at Phu-Cuong commune. In the top-down approach, the local healthcare team and authorities were involved in all screening activities to understand the current cardiac care situation in situ and approve the affordable solutions. In implementation phase, local key persons were also asked to chair IEC campaigns and to receive antihypertensive drugs (if needed). To build up a local cardiac care team, the healthcare staff were equipped with standardised workflows, manuals, guidelines, forms and essential drug stocks for hypertension. The local team was trained and supervised by VNHI doctors, focussing on key procedures such as prescription and adjustment of drugs and health education. VNHI also helped to upgrade cardiac care services at district and provincial level to deal with any difficulties in patient management during the independence and networking phases (Figure 1).

In the bottom-up approach, core IEC messages with some impressive examples of management-related outcomes were broadcast repeatedly to improve local awareness and encourage them to respond more positively to the lifelong check-ups and treatments. Simple and reproducible BP measurements using automatic devices were encouraged so that local people actively witnessed their blood pressure changes and subsequently improved their treatment adherence. In addition, the local cardiac care team tailored the management programme to any emerging demands from the community, reducing support from VNHI until the local staff could run the programme independently.

**Figure 2:**
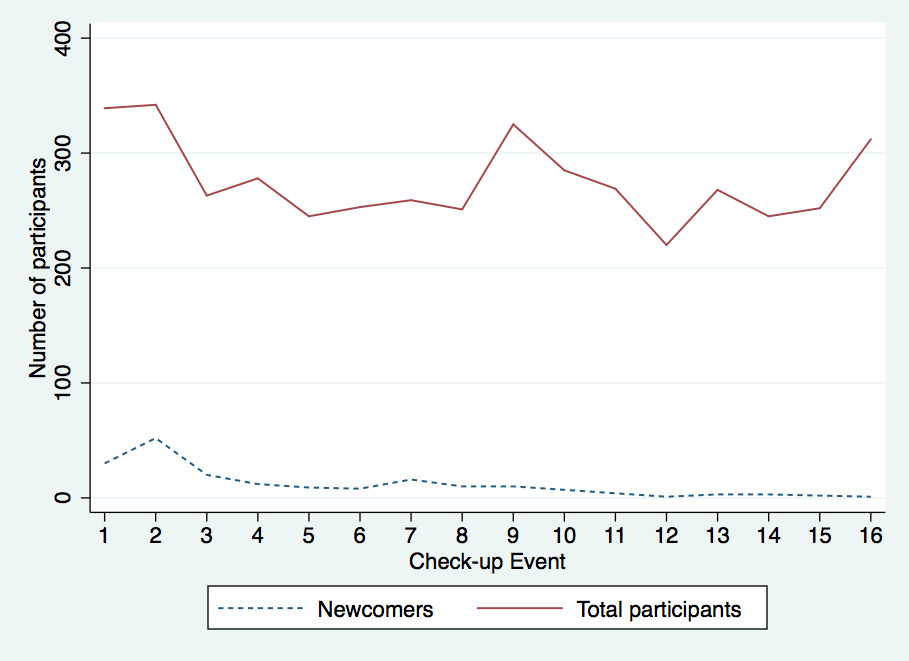
**Rate of newcomer recruitment into the hypertension management programme during 17 months of the implementation phase**.

The programme started with a cross-sectional survey on 1,180 randomly selected adults at Phu-Cuong, which found 469 (39.8%) people with hypertension. Among hypertensives, 37.3% previously knew about their BP, 68.7% did not have any treatment and 0.6% had well-controlled BP. During 17 months of the implementation, the management programme followed up a total of 497 people comprising 38 normotensives (30 people from initial screening & 8 people who joined later) and 459 hypertensives (318 people from initial screening and 141 people who joined later). Overall, 65.6% (326/497) maintained regular follow-up, 20.1% (100/497) had irregular follow-up and the overall dropout was 14.3% (71/497). After the first two months, the number of participants at monthly check-up events was quite stable except for the number of participants at the 9^th ^and 16^th ^check-up events, which related to regular supervision activities and IEC campaigns that were undertaken for the entire community at the same time (Figure 2).

After the screening survey, 67.8% of the identified hypertensives joined the management programme. Those who declined to join the programme were younger and had milder hypertension than people who joined the programme. The former were also more likely to work as manual labourers, have higher educational status and have other chronic diseases or family history of CVD. Characteristics of the third opportunistic group, the group who joined after screening, were similar in terms of age, sex and number of behavioural risk factors or history of other chronic diseases, but a higher proportion suffered from severe hypertension (stage II or III) and they also had a higher mean level of systolic or diastolic BP (Table 1). Using multivariable regression analysis to adjust for demographic variables, only a few variables such as age, initial blood pressure, and a history of cardiovascular diseases were independent predictors for joining the programme, but being female, having several behavioural risk factors and history of other chronic disease were independent predictors for not joining the programme (Table 2).

**Table 1:** General characteristics of hypertensive people, who did not join, joined after screening survey or joined the management programme later

Characteristics	Hypertensives after screening		New participants (n = 141)
		
	Did not join (n = 151)	Joined (n = 318)	
Age (years) (mean ± SD)	54.7 ± 16.1**	65.5 ± 14.1	65.6 ± 12.4

Male (%)	42.4	46.2	48.2

Education status			
*Primary school or lower (%)*	26.5**	45.0 **	-
*Secondary school or higher (%)*	73.5 **	55.0 **	-

Marital status			
*Married (%)*	82.1	75.2	-
*Other (%)*	17.9	24.8	-

Occupation			
*Manual worker (%)*	51.7 **	32.1 **	-
*Other (%)*	48.3 **	67.9 **	-

Distance to health station (km)	0.95 ± 0.48	1.00 ± 0.49	-

Having insurance (%)	35.8	44.0	-
Having voluntary insurance (%)	4.6	3.1	-

Initial systolic BP (mean ± SD)	148.5 ± 16.8 **	163.4 ± 19.4 **	174.5 ± 21.2 **

Initial diastolic BP (mean ± SD)	88.3 ± 9.1 **	92.7 ± 11.6 **	96.7 ± 13.1 **

Initial blood pressure categories			
*Pre-hypertensive stages^+ ^(%)*	0.7	0.6	0.0 **
*Hypertension stage I (%)*	79.5 **	45.9 **	22.0 **
*Hypertension stage II (%)*	12.6 **	32.4	36.9
*Hypertension stage III (%)*	7.3 **	21.1 **	41.1 **

Number of behavioural CVD risk factors	1.0 ± 0.8 **	0.6 ± 0.8	0.7 ± 0.8

Having history of CVD (%)	20.5 **	50.9 **	6.4 **

Having history of chronic disease (%)	16.6 *	10.1	7.1

Having family history of CVD (%)	31.1 **	14.8	17.0

^+ ^Hypertensive people but well treated; * p < 0.05; ** p < 0.01

**Table 2:** Adjusted odd ratios of variables, which deterred people from joining the management programme after screening

Characteristics	Adjusted odd ratio (95% CI)	
	
	Hypertensives (n = 469)	**All people **^**+ **^**(n = 1180)**
Age (by 10 years)	0.67 (0.52 - 0.87)**	0.68 (0.55 - 0.83)**

Sex (female vs. male)	2.14 (1.21 - 3.80) **	1.86 (1.16 - 2.98) **

Education (secondary vs. primary)	1.23 (0.62 - 2.47)	1.25 (0.71 - 2.19)

Occupation (other vs. manual)	1.24 (0.70 - 2.18)	1.01 (0.63 - 1.63)

Married (other vs. married)	1.92 (0.93 - 3.97)	2.11 (1.13 - 3.94) *

Distance to health station (by 1 km)	0.60 (0.37 - 0.97)*	0.77 (0.52 - 1.14)

Having insurance	0.72 (0.42 - 1.22)	0.68 (0.43 - 1.06)

Initial systolic BP (by 10 mmHg)	0.71 (0.59 - 0.86)**	0.50 (0.42 - 0.59)**

Initial diastolic BP (by 10 mmHg)	0.69 (0.53 - 0.92)**	0.60 (0.47 - 0.78)**

Number of behavioural CVD risk factors	1.98 (1.44 - 2.73)**	2.18 (1.65 - 2.88)**

Having history of CVD	0.36 (0.21 - 0.62) **	0.39 (0.24 - 0.63)**

Having history of chronic disease °	2.91 (1.45 - 5.82)**	2.42 (1.28 - 4.57)**

Having family history of CVD	1.86 (1.03 - 3.34) *	1.24 (0.76 - 2.01)

^+ ^Include hypertensive patients; ° exclude CVD history; * p < 0.05; ** p < 0.01

The preliminary data showed that there were significant reductions of systolic and diastolic BP in those having regular follow-ups (55.5/28.7 mmHg) and in those having irregular follow-up (29.2/15.9 mmHg) p < 0.01, but not significantly so in the dropout group (17.6/7.9 mmHg), p > 0.05 (Table 3). However, the more compliant groups received more combined drugs or higher doses to achieve these reductions. Among hypertensives, the dropout rate was significantly higher among mild hypertensives than in the severe hypertensive group (21.5% (38/177) versus 8.2% (23/280) respectively, p < 0.01). During the follow-up period, local staff registered 6 cardiac deaths, 3 non-fatal strokes and 2 heart failures (classified as major adverse cardiac events (MACE)) and 5 minor issues (1 drug-induced cough disappeared after drug replacement, 1 drug-related peripheral oedema disappeared without specific treatment after 2 months, 1 acute pneumonia and 2 unsustained benign arrhythmias). All MACEs happened in patients with chronic and severe hypertension, which perhaps could not be countered with short-term treatments. No adverse drug reactions were registered.

**Table 3:** General characteristics between three levels of adherence to the management programme among hypertensive people

Characteristics	**Drop out**^**§**^ (n = 61)	**Irregular follow-up**^**§**^ (n = 84)	**Regular follow-up**^**§**^ (n = 314)
Age (year) (mean ± SD)	62.6 ± 15.4	63.3 ± 15.5	66.7 ± 12.5*

Male (%)	49.2	50.0	45.5

Education			
*Primary school or lower (%)*	33.3	48.2	46.5
*Secondary school or higher (%)*	66.7	51.8	53.5

Marriage status			
*Married (%)*	84.4	75.0	73.3
*Other (%)*	15.6	25.0	26.7

Occupation			
*Manual workers (%)*	46.7	50.0	24.4 **
*Other (%)*	53.3	50.0	75.6 **

Distance to health station (km)	1.1 ± 0.5	1.0 ± 0.5	1.0 ± 0.5

Having insurance (%)	33.3	41.1	47.0

Initial blood pressure categories			
*Pre-hypertensive stages^+^(%)*	0.0	0.0	0.7
*Hypertension stage I (%)*	62.3	60.7	28.0 **
*Hypertension stage II (%)*	26.2	22.6	38.2 **
*Hypertension stage III (%)*	11.5	16.7	33.1 **

Number of behavioural CVD risk factors	0.8 ± 0.8	0.8 ± 0.8	0.6 ± 0.7

History of cardiovascular diseases (%)	18.0 **	27.4 **	43.6 **
History of other chronic diseases (%)	6.6	7.1	10.2
Family history of CVD (%)	8.2	17.9	16.2

Changes in blood pressure			
*Systolic BP reduction (mmHg)*	17.6 ± 15.6 **	29.2 ± 16.0 **	55.5 ± 21.7 **
*Diastolic BP reduction (mmHg)*	7.9 ± 7.3 **	15.9 ± 8.8 **	28.7 ± 11.9 **
*Hypertension stage changes*	1.1 ± 1.1 **	1.7 ± 1.1 **	3.2 ± 1. 2**

Number of drugs to combine	0.9 ± 0.9 **	1.5 ± 1.0 **	2.5 ± 0.7 **

Number of tablets to take daily	0.9 ± 1.1**	1.4 ± 1.2 **	2.6 ± 1.4 **

Drug side effects or minor diseases (%)	0.0	1.2	1.3

Major adverse cardiac events (%)	3.3	4.8	1.3

^§ ^Adherence to management program: regular: one check-up per 1-2 months; irregular: one check-up per 3-6 months; drop-out: less than one check-up per 6 months.

^+ ^Hypertensive people but well treated; * p < 0.05; ** p < 0.01

Comparing groups of hypertensive patients with different adherence to the management programme, people who dropped out were younger, initially had less severe hypertension, were less likely to have a history of CVD and more likely to be manual labourers (Table 3). Using multivariable regression analysis to adjust for other factors, only a few variables such as changes in blood pressure (either systolic or diastolic BP) and number of combined antihypertensive drugs were independent predictors for programme compliance (Table 4).

**Table 4:** Adjusted odd ratios of variables associated with drop-out from the management programme, comparing the regular follow-up group and the dropout group

Characteristics	Adjusted odd ratio (95% CI)	
	
	Hypertensives (n = 375)	**All people**^**+**^ (n = 397)
Age (by 10 years)	1.32 (0.85 - 2.06)	1.20 (0.81 - 1.79)

Sex (female vs. male)	1.29 (0.35 - 4.78)	1.80 (0.51 - 6.31)

Percentage of systolic BP reduction (by 10%), compared to initial value	0.15 (0.05 - 0.40)**	0.19 (0.08 - 0.47)**

Percentage of diastolic BP reduction (by 10%), compared to initial value	0.27 (0.10 - 0.70)**	0.24 (0.10 - 0.59)**

Number of behavioural risk factors	0.53 (0.21 - 1.31)	0.57 (0.25 - 1.31)

Having CVD history	2.37 (0.53 - 10.69)	2.15 (0.53 - 8.78)

Having chronic disease history°	1.89 (0.26 - 13.84)	1.20 (0.21 - 6.97)

Having family history of CVD	0.36 (0.05 - 2.40)	0.31 (0.06 - 1.67)

Number of tablets to take daily	2.48 (0.81 - 7.61)	2.50 (0.84 - 7.40)

Number of drugs to combine	0.06 (0.01 - 0.25)**	0.06 (0.02 - 0.26)**

Having a minor event	-	-

^+ ^Include hypertensive patients; ° exclude CVD history; * p < 0.05; ** p < 0.01

## Discussion

### Key factors for a hypertension management programme at community level

Emerging non-communicable risk factors (including hypertension) were relatively new concepts to both the local healthcare team and the local population at Phu-Cuong commune, where people were only familiar with traditional infectious diseases. Our experience showed that a convenient infrastructure (including active and effective operations of the local healthcare team), appropriate knowledge of CVD risk factors in the population and an engaged community were the key factors for the successful implementation and maintenance of the hypertension management programme at commune level. Applying both top-down and bottom-up approaches encouraged local people to actively join and comprehensively understand the programme. Engaging the local community as much as possible was crucial for developing new social norms for a heart-healthy environment, to grow the local will for hypertension or other CVD risk factor prevention and then to assure the long-term sustainability of the programme.

In our programme, three dimensions of the conceptual framework for CVD prevention in public health were integrated, which combined an intervention strategy for the population-wide behavioural risk factors (i.e. IEC efforts to reduce BP in community by changes in behavioural risk factors) and an intervention strategy for the high risk group (i.e. efforts to control BP in hypertensives using multiple drugs), using the existing primary care system to carry out health education campaigns for the population, screening for hypertension and management activities for hypertensives [17-20]. In Ba-Vi, the interest in hypertension was fuelled by earlier studies noting a higher rate of awareness and treatment of hypertension in 2005 than in 2002 [12]. However, this motivation itself was insufficient to start a programme without engaging the local community (especially getting support from local authorities) or having additional infrastructure development in place (especially a capable cardiac care team at primary health care level). In late or slow-adopter communities like rural areas of Phu-Cuong (i.e. communities lagging in terms of the receipt of ideas and opportunities), firm support, obvious contributions and live examples from local authority figures (considered as early-adopter individuals) had great impacts on any activities of the programme, especially motivating shifts in the behaviour, knowledge and attitude of the whole population [18,21]. To build up an effective local cardiac care team, on-site hands-on training and continuous on-demand support by the professional team from VNHI were very important. The standardised workflows provided made local staff familiar with new protocols and helped them to reduce accidental errors or any new heavy burden of paperwork. In addition, the co-operation between local healthcare team and VNHI deeply engaged the local team in the programme and also helped them to gain enough confidence for their later practice.

### Factors affecting recruitment and following in the programme

The severity of hypertension and effectiveness of treatment were the main factors influencing the decision of people to join or to leave the programme on hypertension management (Table 2 and 4). These results could be explained by the fact that hypertensives often had to make a trade-off between invisible short-term benefits from prevention and obvious inconvenience from routine check-ups (such as time consumed, difficulties in changing health behaviour, side effects of drugs, feelings of being ill when taking lifelong drugs). Symptoms of hypertension or the burden of its complications became evident usually only at late stages, while these signs were often ignored at the early stages, especially if people were not aware of hypertension and other CVD risk factors. Field experience revealed that it was very hard for a person of working-age with mild hypertension to spend time on check-ups.

Our results interestingly showed that having several behavioural risk factors or a history of chronic diseases were the factors that prevented local people from joining the programme as these patients were usually already treated, although they were not significant factors for people leaving the programme (Tables 2 and 4). Similarly to many developing countries, the prevalence of hypertension was generally lower in women than in men [11,12,22] but it reversed in some socially less privileged groups in Ba-Vi area, such as among women with lower occupational or economic status [11]. Vietnamese women possibly tolerate hardships better and sacrifice more for their families in traditional Confucian-based gender ideologies with a culture of female subordination [23]. Perhaps this tradition tends to make women neglectful of chronic diseases (including hypertension) until complications happen. In Ba-Vi, lower education was associated with hypertension (mostly in males) [11] and with clustered chronic non-communicable disease risk factors [24]. Personal education did not significantly impact on people's decision to join or leave the programme in this study.

The hypertension management programme was able to run independently at the commune health station of Phu-Cuong with a reasonable drop-out rate after 17 months of implementation and benefitted the hypertensives by reducing blood pressure (especially in the regular follow-up group). These preliminary results suggested the feasibility and applicability of the programme in low-resource settings like Phu-Cuong commune.

### Future of the hypertension management programme and implications for health policy makers

The field experiences revealed many difficulties in applying the information from repeated local IEC campaigns to daily life, especially among young males, although quitting smoking and reduced salt diets were cost-effective interventions for reducing blood pressure in the community [19]. Supportive health policies at the top level (such as legislation to ban smoking in public or to reduce the amount of salt in processed foods) would probably be required to make real changes in the community.

In comparison with other chronic diseases, the hypertension management programme had some advantages such as potentially larger benefits (due to high prevalence of hypertension in the community) and easier implementation or replication (due to simple and reproducible methods). In the initial model at Phu-Cuong, apart from behavioural CVD risk factors, high blood pressure was the main target for interventions to reduce CVD burden in the community, especially stroke, which was mostly related to uncontrolled hypertension [25,26] and was the leading cause of death in Vietnam [27]. In a long-term perspective, this programme could be used as a framework to set up new intervention programmes for more advanced CVD or other chronic diseases in community.

Hypertension management at commune level was an important but challenging step in moving essential cardiac care services into primary health care sectors in Vietnam. This step enabled disadvantaged groups (e.g. poor people in rural areas) to approach affordable cardiac care services at convenient places in a timely fashion and to cut down the extra costs of treatment (e.g. transportation fees, expenses for relatives, opportunity cost) thus avoiding the "medical poverty trap" [28-30]. For chronic diseases requiring lifelong treatments such as hypertension, available and well-qualified facilities at primary healthcare level would improve treatment adherence, promote changes of behavioural risk factors and eventually contribute to local secondary and primary prevention activities. But this step challenges the capacity of existing healthcare systems, especially if these systems become overloaded by emerging demands for treatment, although these issues can be overcome by an effective cardiac care network and corresponding supportive health care policies.

## Conclusions

In a rural area, a comprehensive programme of hypertension management at primary healthcare level can be implemented successfully and benefit severe hypertensives. The implementation process required both top-down and bottom-up approaches to engage the whole community and the health care system, supported by committed local authorities and an outstanding professional institute.

## Competing interests

The authors declare that they have no competing interests.

## Authors' contributions

QNN design the study, carried out the project, supervised the screening survey, the implementation of the management programme and drafted the manuscript. STP carried out the project, supervised the screening survey, the implementation of the management program. VLN, SW, LW, RB, PB participated in the design of the study, advised on the implementation process and helped to write the manuscript. All authors have read and approved the final manuscript.

## Pre-publication history

The pre-publication history for this paper can be accessed here:

http://www.biomedcentral.com/1471-2458/11/325/prepub
